# A machine‐learning approach for extending classical wildlife resource selection analyses

**DOI:** 10.1002/ece3.3936

**Published:** 2018-02-28

**Authors:** Kevin T. Shoemaker, Levi J. Heffelfinger, Nathan J. Jackson, Marcus E. Blum, Tony Wasley, Kelley M. Stewart

**Affiliations:** ^1^ Department of Natural Resources and Environmental Science University of Nevada, Reno Reno NV USA; ^2^ Nevada Department of Wildlife Reno NV USA

**Keywords:** habitat suitability, logistic regression, machine learning, *Odocoileus hemionus*, random forest, resource selection function

## Abstract

Resource selection functions (RSFs) are tremendously valuable for ecologists and resource managers because they quantify spatial patterns in resource utilization by wildlife, thereby facilitating identification of critical habitat areas and characterizing specific habitat features that are selected or avoided. RSFs discriminate between known‐use resource units (e.g., telemetry locations) and available (or randomly selected) resource units based on an array of environmental features, and in their standard form are performed using logistic regression. As generalized linear models, standard RSFs have some notable limitations, such as difficulties in accommodating nonlinear (e.g., humped or threshold) relationships and complex interactions. Increasingly, ecologists are using flexible machine‐learning methods (e.g., random forests, neural networks) to overcome these limitations. Herein, we investigate the seasonal resource selection patterns of mule deer (*Odocoileus hemionus*) by comparing a logistic regression framework with random forest (RF), a popular machine‐learning algorithm. Random forest (RF) models detected nonlinear relationships (e.g., optimal ranges for slope and elevation) and complex interactions which would have been very challenging to discover and characterize using standard model‐based approaches. Compared with standard RSF models, RF models exhibited improved predictive skill, provided novel insights about resource selection patterns of mule deer, and, when projected across a relevant geographic space, manifested notable differences in predicted habitat suitability. We recommend that wildlife researchers harness the strengths of machine‐learning tools like RF in addition to “classical” tools (e.g., mixed‐effects logistic regression) for evaluating resource selection, especially in cases where extensive telemetry data sets are available.

## 1 INTRODUCTION

Resource selection functions (RSFs) have tremendous utility for ecologists and resource managers because they enable quantification and prediction of spatial resource‐use patterns by wildlife (Avgar, Potts, Lewis, & Boyce, 2016; Gillies et al., 2006; Johnson, Nielsen, Merrill, McDonald, & Boyce, 2006; Johnson, Seip, & Boyce, 2004). RSFs are typically fitted using a logistic regression framework (commonly, conditional or mixed‐effects logistic regression) to compare a set of relevant environmental features (e.g., topographic, edaphic, or biotic conditions) at “known‐use” resource units (geographic sites known to be selected by focal individuals or populations, often derived from telemetry data) with a set of resource units that are theoretically accessible to the focal individual or population (“available” points; Manly, McDonald, Thomas, McDonald, & Erickson, 2002; Gillies et al., 2006; Johnson et al., 2006). As linear models, classical RSFs possess important limitations; notably, it is often difficult to correctly identify or fit resource selection functions that are not (logit) linear in form, and it is even more challenging to identify complex, nonlinear interactions (Olden, Lawler, & Poff, 2008).

Machine‐learning methods are rapidly gaining traction in ecology because of their ability to overcome the limitations inherent to model‐based inferential methods such as logistic regression (Kampichler, Wieland, Calmé, Weissenberger, & Arriaga‐Weiss, 2010; Olden et al., 2008; Oliveira, Oehler, San‐Miguel‐Ayanz, Camia, & Pereira, 2012). With model‐based inference (e.g., logistic regression), data are used to fit models conceived a priori based on assumptions such as linearity and additivity. In machine‐learning methods such as random forest (RF; Breiman, 2001) and boosted regression trees (BRT; Elith, Leathwick, & Hastie, 2008), a model is elicited from the data, obviating the need for the researcher to impose constraints such as linearity or additivity (Cutler et al., 2007; Olden et al., 2008). Machine‐learning methods free the researcher from imposing strong assumptions and therefore can expose unexpected nonlinear functional relationships and complex interactions in parameter space. This flexibility comes at a price, however, because machine‐learning methods are more data‐hungry and computationally intensive than standard model‐based approaches (with fewer a priori constraints, machine‐learning methods can require more data to detect statistical signals) and lack parameters with simple interpretation (e.g., regression coefficients). Nonetheless, machine‐learning approaches are now commonly used by ecologists for species distribution modeling, general ecological classification and prediction, and more (Crisci, Ghattas, & Perera, 2012; Elith & Graham, 2009; Elith & Leathwick, 2009; Phillips & Dudík, 2008).

Modern wildlife telemetry data, especially derived from automated detectors or from satellite GPS collars (which are often programed to record known‐use locations on an hourly or subhourly schedule), can be tremendously data‐rich and often comprise many thousands of observations (Börger, 2016; Cagnacci, Boitani, Powell, & Boyce, 2010; McKee et al., 2015; Morales et al., 2010; Northrup, Hooten, Anderson, & Witttemyer, 2013; Tomkiewicz, Fuller, Kie, & Bates, 2010). Considering the data richness of modern telemetry studies, wildlife ecologists have been slow to adopt machine‐learning approaches for modeling resource selection. Here, we compare a machine‐learning RSF approach (RF) with a “classical” RSF approach (mixed‐effects logistic regression) to assess the extent to which machine learning can improve upon standard model‐based approaches—specifically, to overcome challenges in identifying and characterizing nonlinear functional relationships and complex interactions. As a case study, we apply both analytical approaches to evaluate seasonal variation in resource selection by a migratory ungulate, the mule deer (*Odocoileus hemionus*). Mule deer, a vital game species and keystone species in many ecosystems that are sensitive to management at landscape scales (Kie, Bowyer, & Stewart, 2003), are a frequent target of habitat improvement efforts in the Great Basin (Wasley, 2004).

Mule deer are widely distributed across many ecosystems throughout western North America, occupying habitats ranging from mature forests in Canada (D'Eon & Serrouya, 2005) to deserts in the southwestern United States (Wallmo, Brownlee, & Lecount, 1981). Mule deer often are migratory and populations have experienced periodic declines throughout their range in recent decades (Monteith et al., 2013; Unsworth, Pac, White, & Bartmann, 1999). Like many other migratory species, mule deer migrate to take advantage of optimal quality and availability of forage that are seasonally available in different areas, to reduce energy expenditure during harsh climatic conditions during winter, or to exploit higher quality forages at high elevations during summer (Garrott, White, Bartmann, Carpenter, & Alldredge, 1987; Nicholson, Bowyer, & Kie, 1997; Wallmo & Geist, 1981). Despite the energetic costs of moving between seasonal ranges, migrants often have access to higher quality and quantity of forage and are often heavier in weight with better nutritional condition than nonmigrants, factors that are strongly linked with fitness components in large mammals (Albon & Langvatn, 1992; Bischof et al., 2012; Gaillard, Festa‐Bianchet, Yoccoz, Loison, & Toigo, 2000; White, 1983). To maintain viable populations and to enhance fitness, mule deer require access to high‐quality forage (Austin & Urness, 1983), water sources (Ordway & Krausman, 1986; Rautenstraunch & Krausman, 1989), cover from harsh weather (Fryxell & Sinclair, 1988; Mysterud & Østbye, 1999; Ordway & Krausman, 1986), and access to productive areas for parturition (Fox & Krausman, 1994; Loft, Menke, Kie, & Bertram, 1987; Riley & Dood, 1984). Because seasonal ranges often differ in quality and availability of forage and other resources, selection of resources to enhance fitness likely differs between seasonal ranges.

We used an extensive GPS telemetry data set for a migratory mule deer population to evaluate seasonal patterns in resource selection using a mixed‐effect logistic regression as well as a random forest (RF) machine‐learning approach (Breiman, 2001). Our dual objectives in this project were to better understand seasonal patterns of resource selection by mule deer and to evaluate the advantages and disadvantages of a machine‐learning approach versus a “classical” approach for addressing this ecological question. From an ecological perspective, we hypothesized that (1) mule deer in their winter range would select for lower elevations, and south‐facing slopes, and habitat types dominated by shrubs, including mountain mahogany (*Cercocarpus ledifolius*), (2) mule deer in their summer range would select habitats at high elevations on moderate slopes, and (3) in both seasons, mule deer would avoid pinyon‐juniper woodlands and would select locations closer to sources of water than predicted at random. Methodologically, we hypothesized that RF would outperform standard model‐based RSFs in cross‐validation and would identify nonlinear functional relationships and complex interactions that would result in strongly contrasting geographic projections of habitat suitability within both summer and winter ranges. More generally, we predicted that RF would enable robust insights about seasonal patterns of resource selection by mule deer that would have been difficult to achieve using standard RSF approaches.

## 2 METHODS

### 2.1 Study area

**Figure 1:**
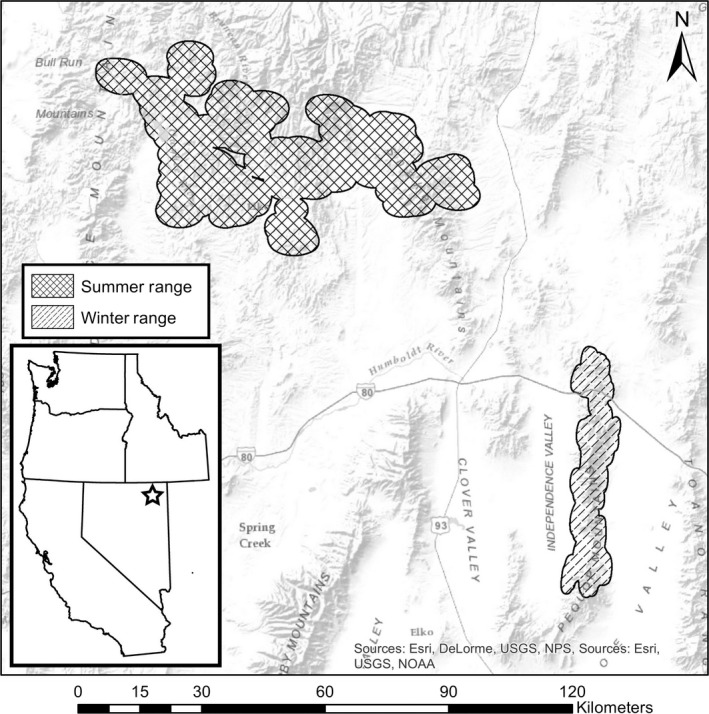
Map of study area, with locations of winter (cross‐hatched region) and summer ranges (dashed diagonal shading) indicated.

Our study area comprised the summer and winter ranges for a migratory population of mule deer in eastern Nevada (Figure 1). In summertime, this population occupied portions of the Jarbidge wilderness in the northeastern corner of Nevada, USA, and the population migrated approximately 170 km south to their winter range in the Pequop Mountains (M.E.B. and T.W., unpublished data). The Jarbidge Wilderness was administered primarily by the U.S.D.A. Forest Service and other portions of summer range were managed privately and by the Bureau of Land Management (Beck, Peek, & Strand, 2006). Dominant vegetation types in the Jarbidge mountains (summer range) included as follows: sagebrush (*Artemisia tridentata*) shrublands, aspen (*Populous tremuloides*) woodland, deciduous shrub communities, desert scrub, grasslands, introduced grasslands, mountain mahogany (*Cercocarpus ledifolius*) shrublands, pinyon (*Pinus monophylla*)‐juniper (*Juniperus occidentalis*) woodland, riparian areas, roads, and alpine meadows. Elevations ranged from about 1,525 to 3,304 m (Beck & Peek, 2001). Most precipitation fell as snow during winter (Beck & Peek, 2001). The winter range in the Pequop Mountains consisted primarily of public land managed by the Bureau of Land Management. Dominant vegetation communities on the winter range in the Pequop Mountains included as follows: sagebrush shrublands, introduced grasslands, mountain mahogany shrublands, pinyon‐juniper woodlands, and riparian areas. During winter months, mean monthly temperatures ranged from a low of 3 to 12°C (Western Regional Climate Center, 2009–2012, M.E.B. and T.W., unpublished data). Elevation ranged from 1,650 to 2,820 m. Precipitation fell primarily as snow in the winter, and snow depths ranged from 13 to 64 cm annually (Western Regional Climate Center, 2009–2012).

### 2.2 Field data collection

Adult female mule deer were captured in January from 2012 to 2014 with a net‐gun fired from a helicopter (Krausman, Hervert, & Ordway, 1985). Each individual was fitted with an Iridium GPS radio‐collar (G2110E, Advanced Telemetry Systems, Isanti, Minnesota, USA). Fix schedules for GPS locations were collected at 1‐day intervals (if more fixes were recorded, we randomly sampled a single fix per day) while animals were on summer and winter ranges. GPS locations recorded during seasonal migration events (migration start and end dates were determined following Blum, Stewart, & Schroeder, 2015) were excluded from further analysis; all remaining GPS locations were assumed to belong to either the summer or winter range.

To define the set of resource units available to this population of mule deer in their summer and winter ranges, we identified 99.9% kernel density isopleths for each range (generated from all known‐use points using Geospatial Modelling Environment version 0.7.4.0; Beyer, 2015) and we augmented these polygons with a 1,500 m (summer) and 1,000 m (winter) buffer. Buffer sizes were selected on the basis of mean daily movement distances for each season. Finally, we generated random points at a 1:1 ratio with used points within the defined winter and summer ranges to characterize resource availability. All the above spatial analyses were performed using ArcGIS (ArcGIS 10.3.1, Environmental Systems Research Institute [ESRI], Redlands, CA, USA).

For all known‐use and available points, we extracted a suite of relevant environmental characteristics: elevation, slope, east to west aspect (sine of aspect in radians), and north to south aspect (cosine of aspect), and distance to water (Table 1). To characterize the biotic and edaphic environment at used and available points, we also extracted dominant vegetation communities using landcover data from LANDFIRE (www.landfire.gov; see above for dominant vegetation communities found in the winter and summer ranges). We selected sagebrush (the most common vegetation community in both the winter and summer ranges) as the reference class (intercept term) for the logistic regression models. Prior to running analyses, we reduced (thinned) the set of predictor variables to reduce multicollinearity, retaining a set of predictor variables that exhibited linear correlations (*r*) of .65 or less (Long et al., 2014; McKee et al., 2015; Stewart, Bowyer, Kie, Cimon, & Johnson, 2002).

**Table 1:** Summary statistics for used and available points for mule deer on summer (*n* = 52 deer) and winter (*n* = 47 deer) ranges in northeastern Nevada, 2012–2014. Sample sizes for summer range for used (*n* = 13,959) and available (*n* = 13,959) points were slightly greater than those for winter range used (*n* = 10,165) and available (*n* = 10,165) points

Variable	Abbreviation	Used points				Available points			
		Mean	*SD*	Min.	Max.	Mean	*SD*	Min.	Max.
Summer range									
Cosine aspect	Cos aspect	1	0.7	0	2	1.07	0.7	0	2
Sine aspect	Sin aspect	1.01	0.71	0	2	1.06	0.71	0	2
Elevation (m)	Elev	2,057	110	1,754	2,745	2,037	158	1,662	2,895
Slope (%)	Slope	12.16	6.74	0	39	10.44	8.05	0	44
Distance to water (m)	Dist.water	737	465	0	2,814	732	532	0.01	3,298
Winter range									
Cosine aspect	Cos aspect	1.01	0.71	0	2	1.08	0.7	0	2
Sine aspect	Sin aspect	1.02	0.71	0	2	1.06	0.7	0	2
Elevation (m)	Elev	2,243	226	1,732	2,772	2,053	238	1,690	2,818
Slope (%)	Slope	18.32	6.63	0	40	13.33	8.89	0	48
Distance to water (m)	Dist.water	2,066	1,887	0	11,379	3,300	2,615	0	13,161

### 2.3 Mixed‐effects logistic regression

We quantified the selection of resources by mule deer females for both summer and winter ranges by fitting mixed‐effects logistic regression models with a use‐availability design (Johnson et al., 2006; Long et al., 2014; Manly et al., 2002), fitted using the “lme4” package in program R v3.4.0 (Bates, Maechler, Bolker, & Walker, 2015; R Core Team 2017) with a binomial error structure and a logit link (Gillies et al., 2006; Long et al., 2014). Among‐individual variation was accommodated with a random intercept term assigned to each individual, which also helped to reduce biases associated with unequal numbers of GPS locations across individuals (Gillies et al., 2006). We evaluated model support using Akaike's Information Criterion AICc (adjusted for small sample size; Burnham & Anderson, 2003; Long et al., 2014). We used the MuMIn package in R (Barton, 2016) to select the top model from among all possible model combinations, with summer and winter ranges evaluated independently (Barton, 2016). All models within 0–2 AICc units of the top model were evaluated for the presence of uninformative parameters (Arnold, 2010); a parameter was considered uninformative if the parameter improved model performance by a negligible amount (ΔAIC < 2; Arnold, 2010; Aho, Derryberry, & Peterson, 2017). We assessed model performance and predictive skill using cross‐validation (see below).

### 2.4 Random forest analysis

We fitted a random forest (RF; Breiman, 2001) model to discriminate between known‐use and available points within summer and winter ranges, respectively (“party” package in R; Hothorn, Hornik, Strobl, & Zeileis, 2010). Random forest is a machine‐learning algorithm that averages the predictions from multiple independent classification or regression trees (De'ath & Fabricius, 2000) into a robust composite predictive model (Breiman, 2001). We used a distribution‐free RF variant (“conditional inference forests”) that performs recursive partitioning using nonparametric permutation tests and that has been shown to reduce bias in variable selection with respect to conventional recursive partitioning methods (Strobl, Boulesteix, Zeileis, & Hothorn, 2007). Our RF model comprised 500 conditional inference trees, with each tree fitted with a random subset of 3% of the data (the low percentage was due to very high temporal autocorrelation in our GPS telemetry data) and each splitting criterion was chosen from a random subset of 3 (out of 5 total) predictor variables. We selected RF settings based on recommendations from the literature (Cutler et al., 2007; Hothorn, Bühlmann, Dudoit, Molinaro, & Van Der Laan, 2005) and trials with a wide range of alternative parameterizations (using cross‐validation to select the settings with the highest predictive accuracy).

The relative importance of predictor variables with respect to resource selection by mule deer was computed as the average degree to which “out‐of‐bag” prediction error for each tree in the forest increased when observation indices for a predictor variable were randomly scrambled (thereby eliminating information content for that predictor variable). Importance values computed using this method therefore account for both main effects and interactions. We assessed model performance and predictive skill using cross‐validation (see below).

Similarly, we ranked the strength of bivariate interactions in the RF model following three steps (following Elith et al., 2008): (1) Each of the two focal predictor variables was divided into 10 bins, resulting in 100 bins in a 2‐D slice of parameter space, and resource selection propensity for each bin was predicted using the random forest model (holding all other predictor variables constant at mean values; Elith et al., 2008), (2) predictions from step 1 (*n* = 100) were treated as the response variable in an additive (no interaction terms) linear model with one free parameter for each of the 10 bins for each focal predictor variable (thereby fitting 20 parameters)—representing an attempt to recover the RF results on the basis of an additive but otherwise unconstrained function of the two focal variables, and (3) the total predictive error (root mean squared error or RMSE) under the additive model from step 2 was computed as an index of relative interaction strength (degree to which the additive model failed to explain the bivariate predictions from the RF model).

### 2.5 Visualizing resource selection patterns

We generated univariate and bivariate partial‐dependence plots to visualize and interpret differences between the two modeling frameworks in apparent functional relationships linking each predictor variable (or combinations of predictor variables) to resource selection propensity by mule deer. To further evaluate the ecological importance of these differences, we generated maps of predicted resource selection propensity for mule deer in their summer and winter ranges (visualized using the “raster” package in R; Hijmans, 2016). To aid in identifying geographic areas differing in projected resource selection propensity between the two modeling approaches, we generated maps of pixel‐by‐pixel difference in resource selection propensity between the two modeling frameworks (positive values signifying areas identified as more suitable by the RF algorithm).

### 2.6 Cross‐validation

To evaluate the predictive skill of both modeling frameworks (RF and logistic regression), we used a rigorous cross‐validation scheme in which we alternately left out all telemetry locations for a single focal deer in our study (treating each individual deer, rather than each telemetry fix, as an independent validation unit). Using this method, we could rigorously assess the generality and predictive power of our models (e.g., Pearson et al., 2014). Predictive skill was measured using the area under the curve (AUC) metric from a Receiver Operating Characteristic (ROC) analysis (Boyce, Vernier, Nielsen, & Schmiegelow, 2002). Although an imperfect measure of performance for presence/background studies (Golicher, Ford, Cayuela, & Newton, 2012; Jiménez‐Valverde, 2012; Lobo, Jiménez‐Valverde, & Real, 2008), AUC provides a useful common currency for comparing predictive performance between the two modeling frameworks (as the two frameworks used the same presence and background locations). We tested for differences in AUC between RF and GLMM analyses using the DeLong test (DeLong, DeLong, & Clarke‐Pearson, 1988).

All statistical analyses were performed using R (R Core Team 2017), and the code for all analyses and graphical visualizations presented herein (and data for running these analyses) are available on GitHub (https://github.com/kevintshoemaker/Mule_Deer_RFvsRSF).

## 3 RESULTS

We captured and collared 53 adult female mule deer across the duration of the study. For the summer range analysis, our generalized linear mixed‐effect models (GLMM) and RF models were fitted using 13,959 known‐use points (GPS locations) from 47 individuals ($\overset{¯}{x}$ = 297 ± 139.41 fixes per animal) in the Jarbidge study area, paired with an equal number of available points. For the winter range analysis, models were fitted using 10,165 use points from 52 individuals ($\overset{¯}{x}$ = 195.48 ± 101.12 fixes per animal) in the Pequop range, again coupled with an equal number of available points (Table 1). In the summer range, mean covariate values for known‐use points exhibited only minor differences from available points, although known‐use points were characterized by slightly steeper slopes on average (Table 1). In the winter range, known‐use points were generally higher in elevation, steeper, and closer to water with respect to available points (Table 1).

### 3.1 Summer range

**Figure 2:**
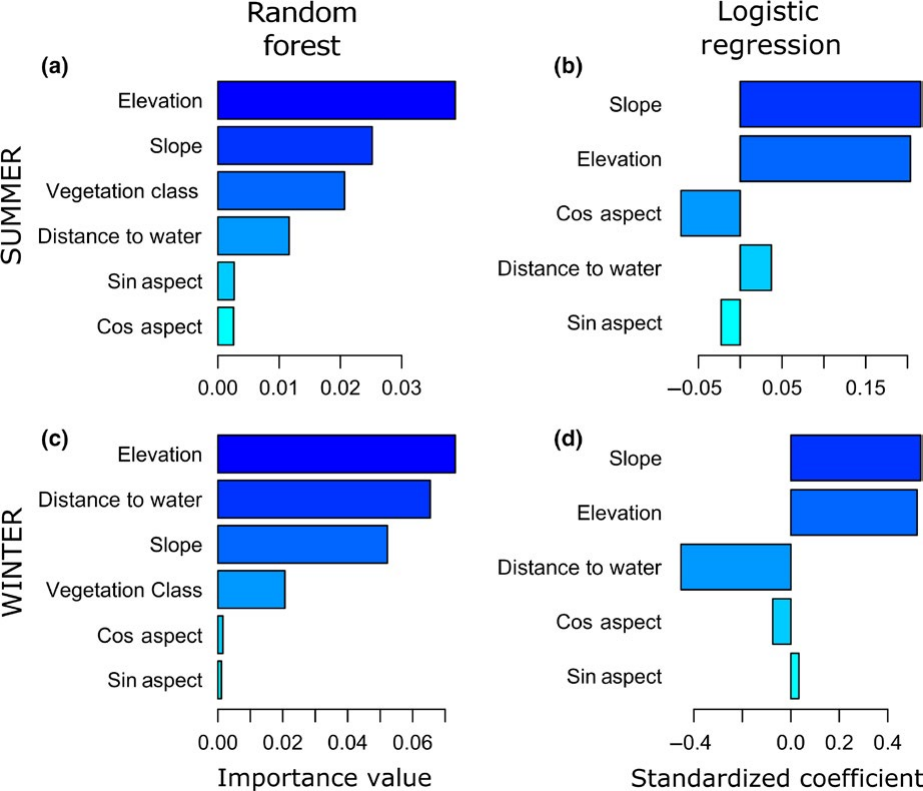
Importance rankings for variables explaining observed seasonal variation in habitat selection patterns derived from (a, c) random forest (RF) and (b, d) generalized linear mixed models (GLMM; “Logistic Regression”) for mule deer in northeastern Nevada, USA, 2012–2014. The top panels represent summer resource selection, and the bottom panels represent winter resource selection. The left panels, derived from RF, depict the overall predictive ability of covariates, accounting for nonlinear responses and interaction effects. The panels on the right, derived from GLMM, depict standardized regression coefficients, which provide an index of the strength of the linear additive relationship for explaining observed habitat selection patterns. Vegetation class is a categorical variable, for which there is no analog to the importance ranking provided by the RF analysis. Note that “Sin Aspect” was deemed uninformative and therefore was not included in the final GLMM models

**Figure 3:**
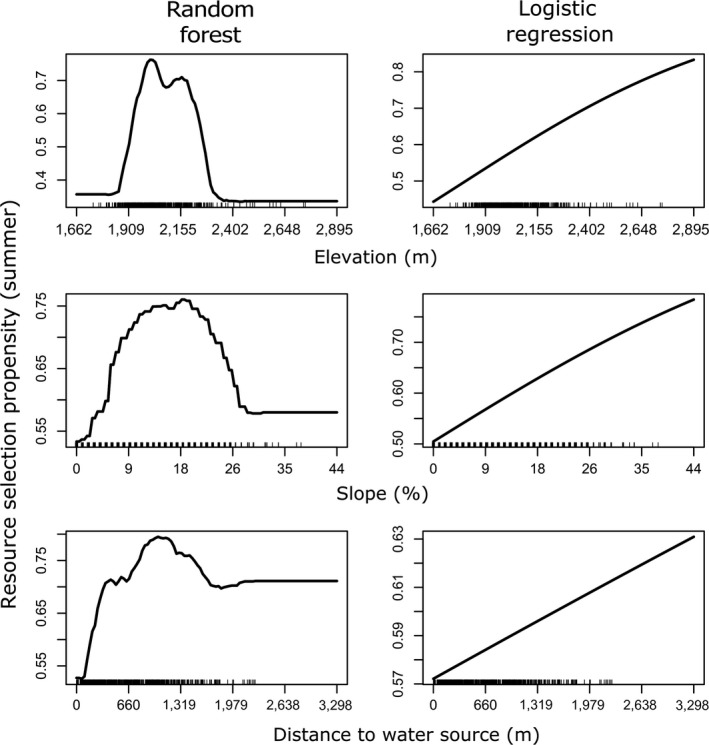
Visualization of key univariate relationships between selected predictor variables and resource selection propensity (proportional to probability of use) by mule deer during summer in northeastern Nevada, USA, 2012–2014. Visualizations were derived from (left column) a random forest model and (right column) a mixed‐effects logistic regression model. The “rug” on the *x*‐axis illustrates the observed distribution of each predictor variable

**Figure 4:**
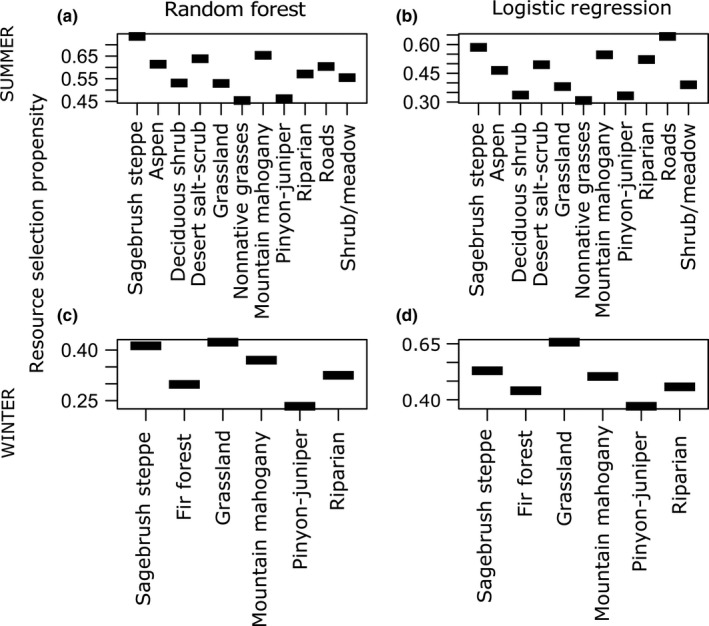
Relative selection propensity of mule deer as a function of dominant vegetation classes, compared across two seasons and two analytical frameworks, in northeastern Nevada, USA, 2012–2014. Top panels (a, b) represent summer habitat selection, and bottom panels (c, d) represent winter habitat selection. Left‐hand panels (a, c) are derived from a random forest analysis, whereas the right‐hand panels (b, d) were derived from a mixed‐effects logistic regression model

The top GLMM (“classical RSF”) model for the summer range included elevation, slope, distance to water, north–south aspect, vegetation class, and interactions terms for (slope × elevation) and (slope × distance to water). East–west aspect and the interaction term between elevation and distance to water were deemed uninformative (Table S1). Results from the GLMM framework indicated that mule deer selected for higher elevations, steeper slopes, and south‐facing slopes in their summer range (Figures 2 and 3). The RF algorithm also identified elevation and slope as key predictors of mule deer habitat use, but south‐facing slopes were not detected as an important driver of resource selection (Figures 2 and 3). Instead, distance to water emerged as a more important predictor in the RF framework, perhaps due to its contribution to interactions in parameter space (see below). Whereas the logistic regression model indicated that the strength of mule deer selection increased across the elevation gradient in the summer range, the RF model detected a highly nonlinear relationship, in which mule deer preferentially selected locations with elevations between ca. 1,900 and 2,250 m (Figure 3). Similarly, whereas the classical RSF indicated that the resource‐use propensity increased across the available gradient of slopes, the RF model indicated that mule deer preferentially occupied slopes between ca. 10 and 25 degrees (Figure 3). The two modeling frameworks yielded similar results for the categorical vegetation class variable (Figure 4). Vegetation classes that were strongly selected included sagebrush steppe, desert scrub, mountain mahogany shrubland, aspen woodland, and sagebrush near roads and development. Mule deer avoided pinyon‐juniper woodland and introduced‐annual grasslands more than the other vegetation communities except for deciduous shrubland (Figure 4).

Both modeling frameworks detected interactions between slope and elevation (Figure 6). The logistic regression model indicated that mule deer in their summer range preferred sites characterized by either steep slopes and low elevations or mild slopes and high elevations. In contrast, the random forest model indicated nearly the opposite that mule deer preferred sites with intermediate slopes and elevations with respect to the available gradient (Figure 6). Similarly, the logistic regression model indicated that mule deer exhibited a preference for high‐elevation sites far from water; in contrast, the RF model indicated that, within the preferred elevation band, mule deer preferentially selected sites that were approximately 0.5–1 km from water (Figure S1).

### 3.2 Winter range

The top GLMM model for the winter range included elevation, slope, distance to water, north–south aspect, vegetation class, and interactions terms for (slope × elevation), (elevation × distance to water), and (slope × distance to water); east–west aspect was deemed uninformative (Table S1). Results from GLMM indicated that mule deer in their winter range generally selected resource units with steeper slopes, higher elevations, and shorter distance to water with respect to available resource units (Figures 2 and 6). The RF algorithm also identified elevation, slope, and distance to water as key predictors of mule deer habitat use in winter, although the order of importance differed from the magnitude of the standardized coefficients from the logistic regression (Figure 2). In strong contrast to the smooth positive relationship identified by the logistic regression model, the RF model detected a highly nonlinear relationship between elevation and the strength of mule deer resource selection, whereby mule deer preferentially selected low elevations (ca. 1,800–2,000 m) or high elevations (>2,400 m), slightly avoiding midrange elevations (Figure 6). The relationship between resource‐use propensity and steepness was similar for the two modeling frameworks, although the RF model indicated that the resource‐use propensity did not increase substantially beyond slopes of ca. 25° (Figure 6). Although both modeling frameworks detected a strong preference for sites close to water, the RF model indicated a strong threshold relationship, with mule deer strongly selecting sites within 1 km of water, with the relationship breaking down past approximately 2 km (Figure 6). Vegetation classes that were strongly selected for in winter included annual grasslands and sagebrush steppe. Mule deer avoided pinyon‐juniper woodlands more than the other vegetation communities in their winter range (Figure 4).

**Figure 5:**
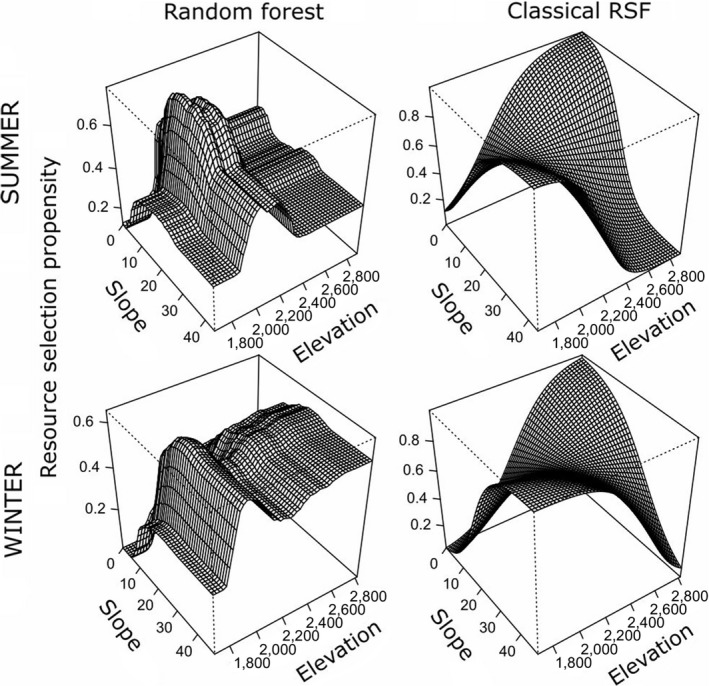
Visualization of habitat selection propensity by mule deer in northeastern Nevada, 2012–2014, as a function of elevation and slope (bivariate partial‐dependence plots), illustrating seasonal differences and differences between alternative analytical approaches. Figures were derived from (left panels) a random forest model and (right panels) a generalized linear mixed‐effects model (“Logistic Regression”). Top panels represent resource selection in the summer range, and lower panels represent resource selection in the winter range

Like the summer range analysis, both frameworks detected a strong interaction between slope and elevation (Figure 5). Mirroring the summer range analysis, the GLMM indicated that mule deer in their winter range preferred sites characterized by either steep slopes and low elevations or mild slopes and high elevations. In contrast, the RF model indicated that mule deer preferred sites that combined intermediate‐to‐steep slopes with intermediate‐to‐high elevations (Figure 5). Both modeling frameworks agreed that mule deer on their winter range exhibited a preference for high‐elevation sites close to water sources. However, the RF model indicated that mule deer tended to avoid high‐elevation sites far from water, whereas the logistic regression model indicated a moderate preference for such sites (Figure S1).

### 3.3 Geographic projections of resource selection propensity

Differences in the shapes of univariate responses and interactions between the two modeling frameworks resulted in strong differences in projected habitat selection propensities across geographic space for both seasonal ranges (Figure 7). For example, a region in the northwest quadrant of the summer range (box A in Figure 7), deemed highly suitable under the GLMM framework, was predicted to be unsuitable under the RF framework. This difference was primarily driven by differences in the slope‐by‐elevation interaction between the two frameworks, whereby low elevation, steep terrain was strongly preferred in the GLMM model and avoided in the RF model (Figure 5). We identified a similar difference in the winter range (box B in Figure 7), whereby a strong difference between RF and GLMM could be primarily attributed to differences in the shape of the slope‐by‐elevation interaction.

### 3.4 Model performance

Predictive skill was strong for both modeling frameworks in the winter range, although RF exhibited slightly improved performance relative to the GLMM framework; rigorous cross‐validation analyses returned AUC values of 0.82 and 0.79 for random forest and mixed‐effects winter range models, respectively (DeLong's test: *D* = −6.913, *df* = 40448, *p*‐value = 4.8e‐12). Predictive skill was much lower in the summer range model for both modeling frameworks, although the RF model again statistically outperformed the GLMM; cross‐validation of the RF analysis for the summer range returned an AUC of 0.67, whereas the AUC from the GLMM analysis was 0.62 (DeLong's test: *D* = −10.64, *df* = 55718, *p*‐value < 2.2e‐16).

## 4 DISCUSSION

The two alternative frameworks that we used to model resource selection patterns in mule deer—mixed‐effects logistic regression and a machine‐learning approach (RF)—yielded some similar results (e.g., stronger selection for water sources in winter; see below). However, we also detected marked differences in apparent functional relationships between the two frameworks. Notably, RF identified some complex nonlinear relationships that would have been difficult to detect using standard methods (e.g., RF identified lower and upper preference bounds for elevation and steepness within the summer range and a bimodal relationship with elevation in the winter range). In several cases, the two modeling frameworks yielded two‐way interactions with strongly opposing interpretations. These differences were ecologically meaningful, resulting in strongly divergent predictions regarding the locations of highly suitable habitat for both the summer and winter ranges. Furthermore, the RF model consistently outperformed the model‐based approach; while this result is not unexpected when comparing an unconstrained machine‐learning algorithm with a constrained model‐based approach (Olden et al., 2008), our cross‐validation results (in which all known‐use locations for individual deer were excluded in turn for model validation) lends support to the conclusion that the increased performance of RF was not an artifact of overfitting.

**Figure 6:**
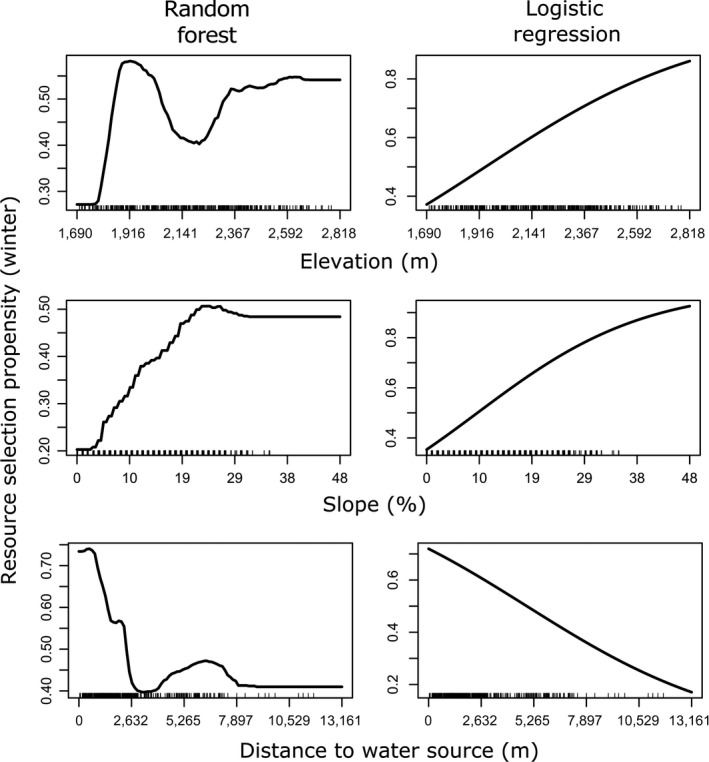
Visualization of key univariate relationships between selected predictor variables and resource selection propensity (proportional to probability of use) by mule deer during winter in northeastern Nevada, USA, 2012–2014. Visualizations were derived from (left column) a random forest model and (right column) a mixed‐effects logistic regression model. The “rug” on the *x*‐axis illustrates the observed distribution of each predictor variable

When two different modeling frameworks agree on the importance and functional relationship between strength of selection and an environmental variable or feature, the combined results can be confirmatory (more convincing than either analysis run independently). For example, classical model‐based analysis can confirm the statistical integrity of the result, and the machine‐learning results can confirm the shape of key functional responses. For example, in our study, the two modeling approaches confirmed the general importance of slope for habitat selection by mule deer in the winter range, and the functional form of that relationship (strong linear increase in use propensity with increasing steepness on the winter range; Figure 6). In addition, the two analyses tended to confirm the propensity to select or avoid each of the dominant vegetation communities available to mule deer in our study; for example, both frameworks indicated that mule deer exhibited a stronger selection preference for annual grasses in the winter range versus the summer range. Finally, both modeling frameworks showed that mule deer exhibited a strong preference for sites near to water in the winter, whereas in summer, distance to water exerted a far lesser influence on habitat selection. The lack of strong tendency to remain close to water in summer seems counterintuitive, because summer lactation increases water requirements for females (Perkins, Smith, & Mautz, 1998). However, the winter range (Pequop mountains) lies in the rain shadow of the Ruby Mountains in eastern Nevada and has a substantially drier climate than the summer range (Jarbidge mountains; Table 1). Thus, mule deer in our study population were likely forced to remain close to scarce water resources in their winter range.

Additional ecological insights can be gained when the two frameworks disagree (as was often the case in our mule deer study). In many instances, the two frameworks agreed about the general importance of a variable, but the machine‐learning approach suggested a nonlinear response. For example, the RF analysis suggested that the relationship between mule deer selection propensity and topographic variables (e.g., elevation) exhibited nonlinear (humped or bimodal) functional forms (Figures 3 and 6). Interestingly, the strong bimodal response to elevation detected by the winter range RF model (Figure 6) likely reflects an avoidance of the elevational zone dominated by pinyon‐juniper woodlands (Bender, Boren, Halbritter, & Cox, 2013). In addition, the RF results suggested a threshold relationship between habitat selection and distance to water in the winter range (Figure 6). Such discrepancies can provide evidence for a violation of the common assumption of logit‐linear functional responses (Boyce & McDonald, 1999). The ability of machine‐learning approaches to accommodate such nonlinearities likely contributed to improved performance of the RF algorithm in cross‐validation. To further confirm the nonlinear functional form (i.e., that it is not an artifact of overfitting), researchers may choose to incorporate a quadratic or threshold term to accommodate putative nonlinearities within a model‐based inferential framework and assess whether these new parameters hold up to statistical scrutiny (e.g., using *p*‐values or AIC model selection).

In instances where a predictor variable is identified as important in a constrained model‐based analysis, but a machine‐learning alternative fails to confirm this finding, this discrepancy can raise doubts about whether the variable is important. Like other machine‐learning alternatives, RF is an unconstrained algorithm and as such can be prone to overfitting, especially with large data sets (Olden et al., 2008). However, if an unconstrained machine‐learning algorithm fails to detect a pattern that was detected by a constrained analysis (e.g., logistic regression) in the presence of a rich data set, the detected pattern may be an artifact of the imposed constraints (e.g., explaining variation that otherwise could have been explained by other variables, either via nonlinear functional forms or complex interactions). For example, in our study, the mixed‐effects logistic regression analysis suggests that mule deer preferentially selected south‐facing slopes during summer and (to a lesser extent) winter (determined based on the “cosine of aspect” variable). This statistical result (*p *<<< .01) might suggest avoidance of colder, north‐facing slopes to reduce thermoregulatory energetic demands, and (in winter) avoidance of deep snow packs. However, the RF analysis failed to confirm this result, suggesting that the preference for south‐facing slopes may be a statistical artifact.

Importance values are a key advantage of the machine‐learning approach, and for which there is no perfect analogy for model‐based statistical approaches (but see Avgar, Lele, Keim, & Boyce, 2017). In the RF modeling framework, importance scores reflect the degree to which each predictor variable contributes useful information for (in the case of resource selection models) discriminating between known‐use and available spatial units (Breiman, 2001; Strobl, Boulesteix, Kneib, Augustin, & Zeileis, 2008). The RF importance values account for nonlinear responses and interactions (even deep multivariate interactions). With model‐based RSFs, standardized regression coefficients can be treated as a measure of relative importance (e.g., Cross & Beissinger, 2001; Menard, 2011; Murray & Conner, 2009). However, this ranking method only account for univariate additive effect size, and in addition fails to holistically account for nonlinear responses. The cumulative AIC model weight, computed as the sum of AIC weights for the subset of models containing the covariable of interest, is also commonly used as measure of ranking relative importance of predictor variables (Burnham & Anderson, 2001; Murray & Conner, 2009). However, this method suffers from some of the same problems mentioned above (fails to account for nonlinearities) and introduces new issues (difficult to account for interaction terms). Therefore, we favor RF and similar machine‐learning approaches as a robust, holistic, and straightforward method for ranking variable importance.

**Figure 7:**
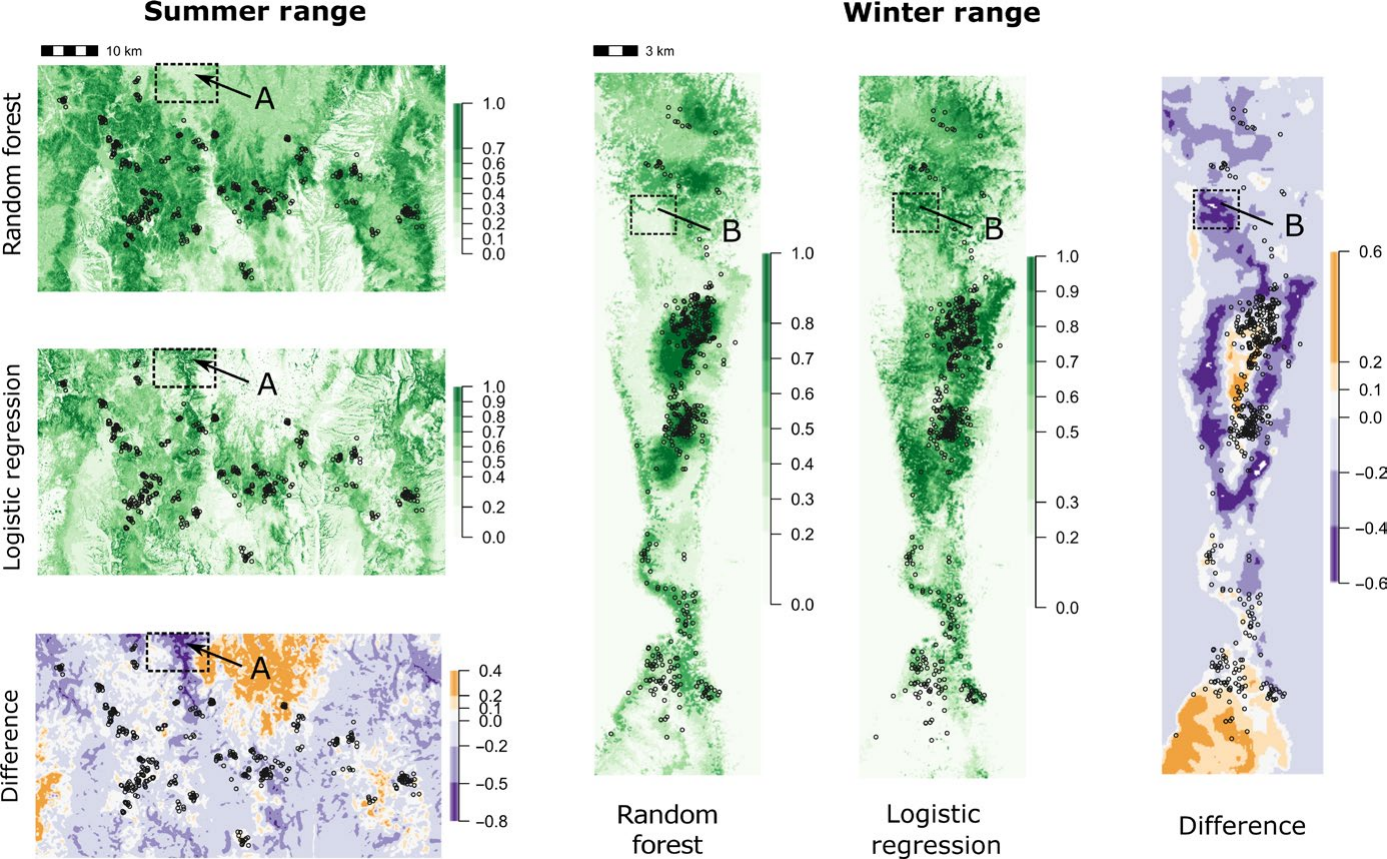
Maps of relative resource selection propensity for a focal population of mule deer in their winter and summer ranges, as projected based on a machine‐learning model (random forest; RF) and a generalized linear mixed‐effects model (logistic regression). “Difference” maps represent differences in projected habitat suitability between the two modeling approaches, with higher values representing areas deemed more suitable under the RF approach. Regions enclosed with dashed rectangles and labeled “A” (summer range) and “B” (winter range) represent areas with strong projected differences that are discussed further in the main text

In our study, perhaps the most striking differences between the two modeling paradigms emerged when visualizing key two‐way interactions in parameter space (Figures 5 and S1). While both frameworks detected strong interactions between the topographic predictor variables (slope, elevation, and distance to water), the shapes of those interactions differed markedly in almost all cases. For example, whereas the GLMM detected a preference for steep, low elevation terrain or shallow, high‐elevation terrain on the summer and ranges, RF indicated avoidance of such areas on the summer and (to a lesser extent) winter ranges (Figure 5). Similarly, a counterintuitive result from the GLMM—that mule deer preferentially selected sites that were high in elevation and far from water sources in the summer range—was contradicted by the RF analysis, which detected a preference for midelevation sites relatively close to water sources (Figure S1). Notably, many of the differences in projected resource suitability between the two frameworks (e.g., boxes A and B in Figure 7) could be attributed to differences in the shapes of key interactions. Overall, it appears that the constraints inherent to standard model‐based RSF methods can present critical barriers for assessing the nature of interactions in parameter space (especially between two continuous variables), and this fact alone should motivate the use of machine‐learning methods alongside classical methods for evaluating patterns of resource selection by wildlife.

While the observation that these two modeling approaches yielded substantially different results can be unsettling, we contend that important insights can be gained from running model‐based resource selection analyses together with machine‐learning alternatives—which require negligible additional work once the data have been prepared for analysis. Unlike machine‐learning approaches, model‐based approaches yield performance benchmarks (e.g., confidence intervals), interpretable coefficients and are amenable to well‐established information‐theoretic approaches for variable selection and multi‐model inference (Boyce et al., 2002). On the other hand, machine‐learning frameworks like RF (also boosted regression trees, neural networks, maximum entropy; Elith & Graham, 2009; Elith & Leathwick, 2009; Olden et al., 2008; Phillips & Dudík, 2008) are far more flexible, do not require a priori specification of nonlinear relationships and interactions, and can therefore help develop a more realistic understanding of how individuals respond to available environmental gradients. In addition, machine‐learning methods can help in identifying and interpreting key interactions, allowing researchers to learn how fundamental resource selection patterns may change across a multi‐dimensional parameter space. Taken together, it may be possible to harness the strengths of both frameworks to build a more robust understanding of habitat use patterns and ultimately aid wildlife researchers and resource managers in identifying and protecting critical habitat.

## CONFLICT OF INTEREST

None declared.

## AUTHOR CONTRIBUTIONS

KMS and TW obtained funding and oversaw the project. MEB collected data for the project in the field. LJH, NJJ, and MEB are graduate students and KMS is graduate advisor. KTS, LJH, and NJJ conceived the idea for the paper as a final class project (KTS was instructor). KTS, LJH, NJJ, and MEB analyzed the data. KTS, KMS, LJH, and NJJ interpreted the results and wrote the manuscript. All authors provided editorial assistance.
