# Supplementary material for: A machine‐learning approach for extending classical wildlife resource selection analyses
Source: Ecol Evol. 2018 Feb 28;8(6):3556–69. doi: 10.1002/ece3.3936 (PMC5869366; doi:10.1002/ece3.3936)
Supplement: Supplementary file 1 [file ECE3-8-3556-s001.docx]

Table S1. Ranked models for resource selection functions during summer for 47 mule deer in the Jarbidge Mountains in northeastern Nevada, 2012-2014. Abbreviations for covariates are described in table 1 with the exception of vegetation class (veg class).

| Model | DF | AICc | ΔAICc | ω |
| --- | --- | --- | --- | --- |
|  |  |  |  |  |
| Cos Aspect + Sin Aspect + Dist. Water + Elev + Slope + Veg Class + (Dist. Water * Slope) + (Dist. Water*Elev) + (Elev * Slope) | 20 | 36548 | 0 | 0.422 |
| Cos Aspect + Sin Aspect + Dist. Water + Elev + Slope + Veg Class + (Dist. Water * Slope) + (Elev * Slope) | 19 | 36549 | 1.133 | 0.239 |
| Cos Aspect + Dist. Water + Elev + Slope + Veg Class + (Dist. Water * Slope) + (Dist. Water*Elev) + (Elev * Slope) | 19 | 36549 | 1.326 | 0.217 |
| Cos Aspect + Dist. Water + Elev + Slope + Veg Class + (Dist. Water * Slope) + (Elev * Slope) | 18 | 36551 | 2.496 | 0.121 |
| Sin Aspect + Dist. Water + Elev + Slope + Veg Class + (Dist. Water * Slope) + (Dist. Water*Elev) + (Elev * Slope) | 19 | 36578 | 29.94 | 0.000 |

Table S2. Ranked models for resource selection functions during winter for 52 mule deer in the Pequop Mountains in northeastern Nevada, 2012-2014. Each model includes an intercept. Abbreviations for covariates are described in table 1 with the exception of vegetation class (veg class).

| Model | DF | AICc | Δ AICc | ω |
| --- | --- | --- | --- | --- |
|  |  |  |  |  |
| Cos Aspect + Sin Aspect + Dist. Water + Elev + Slope + Veg Class + ( Dist. Water*Slope) + ( Dist. Water*Elev) + (Elev*Slope) | 15 | 22153 | 0 | 0.548 |
| Cos Aspect + Dist. Water + Elev + Slope + Veg Class + ( Dist. Water*Slope) + ( Dist. Water*Elev) + (Elev*Slope) | 14 | 22155 | 1.884 | 0.214 |
| Cos Aspect + Sin Aspect + Dist. Water + Elev + Slope + Veg Class + ( Dist. Water*Elev) + (Elev*Slope) | 14 | 22155 | 2.303 | 0.173 |
| Cos Aspect + Dist. Water + Elev + Slope + Veg Class + ( Dist. Water*Elev) + (Elev*Slope) | 13 | 22157 | 4.253 | 0.065 |
| Sin Aspect + Dist. Water + Elev + Slope + Veg Class + ( Dist. Water*Slope) + ( Dist. Water*Elev) + (Elev*Slope) | 14 | 22170 | 16.819 | 0.0001 |


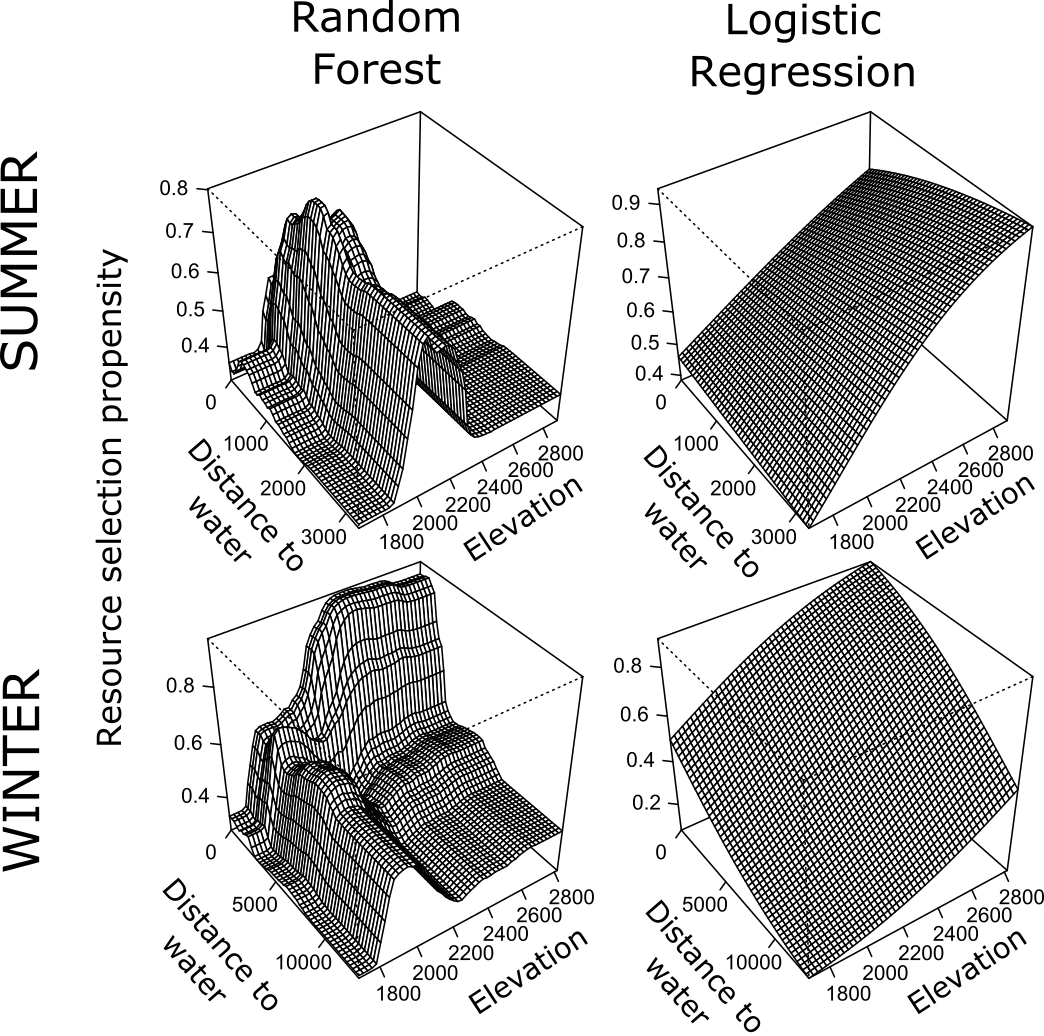


Figure S1. Visualization of habitat selection propensity by mule deer in northeastern Nevada, 2012-2014, as a function of elevation and distance to water (bivariate partial-dependence plots), illustrating seasonal differences and differences between alternative analytical approaches. Figures were derived from (left panels) a random forest (RF) model and (right panels) a generalized linear mixed-effects model (GLMM; “Logistic Regression”). Top panels represent resource selection in the summer range and lower panels represent resource selection in the winter range. Note that the interaction term between elevation and distance to water was deemed uninformative and therefore was not included in the final GLMM model for the summer range.
